# A national survey of Ixodidae ticks on privately owned dogs in Italy

**DOI:** 10.1186/s13071-018-2994-2

**Published:** 2018-07-16

**Authors:** Maria Paola Maurelli, Paola Pepe, Liliana Colombo, Rob Armstrong, Elena Battisti, Maria Elena Morgoglione, Dimitris Counturis, Laura Rinaldi, Giuseppe Cringoli, Ezio Ferroglio, Stefania Zanet

**Affiliations:** 1 Department of Veterinary Medicine and Animal Production, University of Naples Federico II, Naples, Italy; 2 MSD Animal Health, Milan, Italy; 3 MSD Animal Health, Madison, NJ USA; 4 Department of Veterinary Sciences, University of Turin, Turin, Italy

**Keywords:** Ticks, Dogs, Epidemiology, Risk factors, Seasonal distribution, Italy

## Abstract

**Background:**

The geographical distribution of ticks on companion animals needs to be monitored to develop and plan effective control measures, as suggested by the European Scientific Counsel on Companion Animal Parasites. The aim of this study was to conduct the first Italian national survey of tick distribution on privately owned dogs.

**Methods:**

The study was performed over 20 months (February 2016 - September 2017) and involved 153 veterinary practices in 64 different provinces covering 17/20 (85%) Italian regions. Participating practitioners were asked to examine five different dogs per month at random and complete a questionnaire for each dog. Differences in tick infestation associated with: sex, age and hair length (long and short); the dog’s habitat (indoor or outdoor/kennel); and the dog’s environment (urban or rural/sylvatic) were evaluated. The attachment site of ticks on the dog was also recorded. Acaricide efficacy was evaluated for the subset of dogs for which complete information on product used, date of sampling and date of last ectoparasiticide treatment was available.

**Results:**

Of the 3026 dogs examined, 1383 (45.7%) were carrying at least one tick. Overall, 2439 tick samples were collected and a total of 14 tick species identified. *Rhipicephalus sanguineus* group were the most predominant ticks (63.6%), followed by *Ixodes ricinus* (30.6%) and *I. hexagonus* (5.6%). Twenty-four dogs had mixed tick infestations. Long-haired dogs had a higher tick infestation risk as did dogs with outdoor and rural/sylvatic lifestyles. Ticks were located on the head (37.4%), the neck (28.8%), the muzzle (15.5%) and the back (15.3%). A higher prevalence of *Rhipicephalus* was found in the interdigital spaces (10.8%) compared to *Ixodes* (0.2%). Finally, ectoparasiticide treatments were found significantly protective against tick infestation, especially orally administered formulations.

**Conclusions:**

Privately owned dogs in Italy have a high prevalence (45.7%) of infestation with ixodid ticks and this risk varies by dog phenotype and lifestyle.

## Background

In Animal Planet’s list of the top ten most extreme bloodsuckers on Earth, mosquitoes were sixth, leeches fifth, kissing bugs fourth, bedbugs third, fleas second and ticks first [1, 2]. The literature reports either mosquitoes or ticks as the most important vectors of pathogens to animals and humans [2]. Hard ticks (Ixodidae) are ectoparasites of domestic and wild animals, as well as humans. Their medical and veterinary importance is increased by their great capacity for transmitting viral, bacterial, protozoan and helminthic infections to animals, causing a diverse range of infections commonly referred to as tick-borne diseases (TBDs) [3]. Although chemical tick control options are available, the worldwide incidence of human TBDs is increasing. In Europe, an estimated average of 85,000 people are diagnosed every year with Lyme borreliosis [3]. The incidence of human TBDs increased in Italy over the last decade, with 4604 clinical cases and 33 deaths documented by the Ministry of Health in the period 1998–2002, mainly in southern and insular regions [3].

Tick geographical distribution and abundance are influenced by biotic and abiotic factors, such as climate, altitude, urbanization and host population dynamics [4]. In recent years, environmental changes, and the movement of people and animals have introduced novel vector species into previously free areas, leading to changes in local epidemiology of ticks and their associated pathogens [5, 6]. Reducing and controlling these ectoparasites is extremely difficult. Currently, control measures are mainly based on use of chemicals on animals and in the environment [7]. For the multiple reasons mentioned above, it is crucial to develop and implement a systematic surveillance system, based on a comprehensive knowledge of tick species present in a target geographical area [8, 9]. Companion animals, especially dogs, may be useful sentinels for monitoring tick population distribution and also the pathogens they carry [10, 11]. Many national surveys have been conducted in Europe, based on this assumption, to measure tick abundance on dogs and to understand their spatial distribution [9, 10, 12–14]. Italy is a European country with many species of ticks, with about 40 different species reported [3]. However, to date, there has been no surveillance program in Italy, and information regarding Italian ixodofauna of dogs is limited to local surveys of single tick species and their pathogens [15], or to ticks collected from the environment [16–20].

The aim of this study was to conduct the first national Italian survey of ticks in privately owned dogs presented to veterinary practices and to develop a spatial distribution framework of different tick species. Risk factors associated with tick infestation, seasonality and acaricide efficacy were recorded and analyzed.

## Methods

### Study design and tick collection

A nationwide survey was performed in Italy from February 2016 to September 2017. The project involved voluntary participation of veterinary practices in different areas of Italy. The dogs enrolled in the study were homogeneously distributed across the regions using a criterion of proportional allocation, i.e. each region was assigned a number of dogs proportional to the total number of dogs registered in each region [21]. Each veterinary practice was provided with a “tick survey package” containing the study protocol, 60 questionnaires, 60 sample vials with 70% ethanol and a tick removal hook. The protocol instructed participating vets to examine at least five dogs per month for ticks over the 20-month study period. Dogs were to be randomly chosen without prior knowledge of their tick infestation status. The questionnaire requested information on the location of the sampled dog (post code of the owner or, if this was not given, the practice postcode), breed, sex, age, hair length and recent ectoparasiticidal treatment (the last treatment for ticks and the drug used). Additional questions included: the type of housing (indoor or outdoor/kennel), the environment in which dog is usually moved (urban, rural/sylvatic), and the attachment site of ticks if present.

At the time of the visit, each dog was thoroughly visually examined for 15–20 min to detect any ticks present. Eleven body regions were observed including: head, muzzle, neck, armpits, back, abdomen, arts, tail, anus, vagina and interdigital spaces. All ticks found were removed and preserved in the sample vials at room temperature for submission to the laboratories of Parasitology in Turin and Naples. An individual identification number (ID) was assigned to each animal and questionnaires and vials were labeled with the same ID.

### Data handling and tick identification

Questionnaire and sample vial data were entered into an Excel (Microsoft, Redmond, WA, USA) spreadsheet. Ticks were identified to species level, life-stage (i.e. larva, nymph or adult) and gender (female or male) under a stereomicroscope using appropriate morphological keys [22–24].

Specimens (86) that were difficult to identify from morphological characterization were selected for genetic analysis. DNA was extracted using a commercial kit (HiPurA™ PCR Product Purification Kit, HiMedia, Mumbai, India) in accordance with the manufacturer’s instructions. Partial mitochondrial *12S* rRNA and *16S* rRNA gene sequences were generated and analyzed using primers and PCR conditions as previously described [24, 25]. Amplicons were resolved in ethidium bromide-stained (1.5%) agarose gels (Bio-Rad, Madrid, Spain) and sized by comparison with a marker in the 6X DNA Loading Dye (Thermo Fisher Scientific, Waltham, USA). Gels were photographed using a digital documentation system (Gel Doc 2000, Bio-Rad, Watford, UK). PCR products (amplicons) were purified (HiPurA™ Mammalian Genomic DNA Purification Kit, HiMedia) and sequenced. Sequences were analyzed using the Chromas version 2.1.1 software and compared with the partial mitochondrial *12S* and *16S* rRNA gene sequences in GenBank.

### Mapping and statistical analysis

The location of positive dogs for each tick genera was geo-referenced using a geographical information system (GIS, ArcGIS version 10.3 ESRI), referring to the owner’s postcode or, if missing, to the veterinary practice postcode for the dog. GIS was also used to analyze spatial information on body locations of ticks on examined dogs.

Tick infestation differences were analyzed in association with dog sex, age, hair length (long and short), habitat (indoor or outdoor/kennel) and environment (urban or rural/sylvatic) using the Chi-square test, with presence or absence of ticks as dependent variable. Four categories were used for dog gender: male, female, male neutered and female neutered. Dogs were classed into five groups for the age analysis: puppies (less than 1 year of age); young-adult (1–3 years); adult (4–6 years); old (7–10 years); and very old (> 10 years). Acaricide efficacy was evaluated for those dogs for which complete information on the product used, date of sampling and date of last acaricide treatment was available. Differences were considered significant at *P* < 0.05. Chi-square tests and logistic regression were performed using SPSS Statistics 20.0 (IBM Corp. Released 2011. IBM SPSS Statistics for Windows, Version 20.0. Armonk, NY, USA).

## Results

**Fig. 1:**
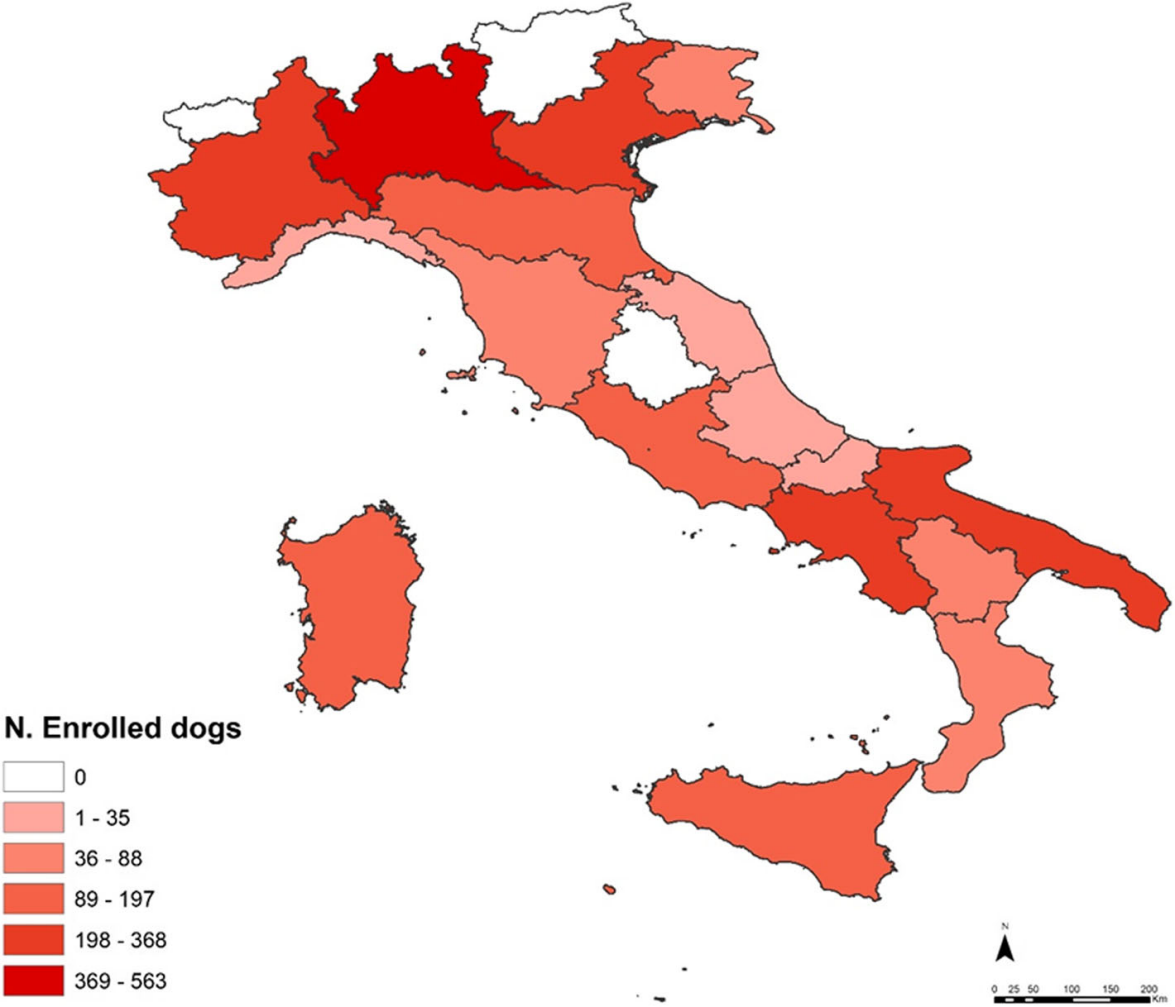
Dogs enrolled in a survey of tick infestation in Italy showing the frequency of dog enrollment for each Italian region

A total of 153 veterinary practices from 64 different provinces of 17/20 (85.0%) regions of Italy (Fig. 1) participated in the survey and 3026 dogs from northern (1520), central (283) and southern (1223) Italy were examined and had a completed questionnaire. The enrolled dogs included: 835 intact (27.6%; 95% CI: 26.0–29.2%) and 562 neutered females (18.6%; 95% CI: 17.2–20.0%), 1430 intact (47.3%; 95% CI: 45.5–49.1%) and 199 neutered males (6.6%; 95% CI: 5.7–7.5%). The age of animals ranged from 1 month to 17 years (median age of 4 years). The dogs belonged to over 100 different breeds, of which cross-bred were the most prevalent (39.3%; 95% CI: 37.5–41.0%). Most had short hair (56.6%; 95% CI: 54.9–58.4%) and were housed rather than kenneled (57.2%; 95% CI: 55.5–59.0%).

**Fig. 2:**
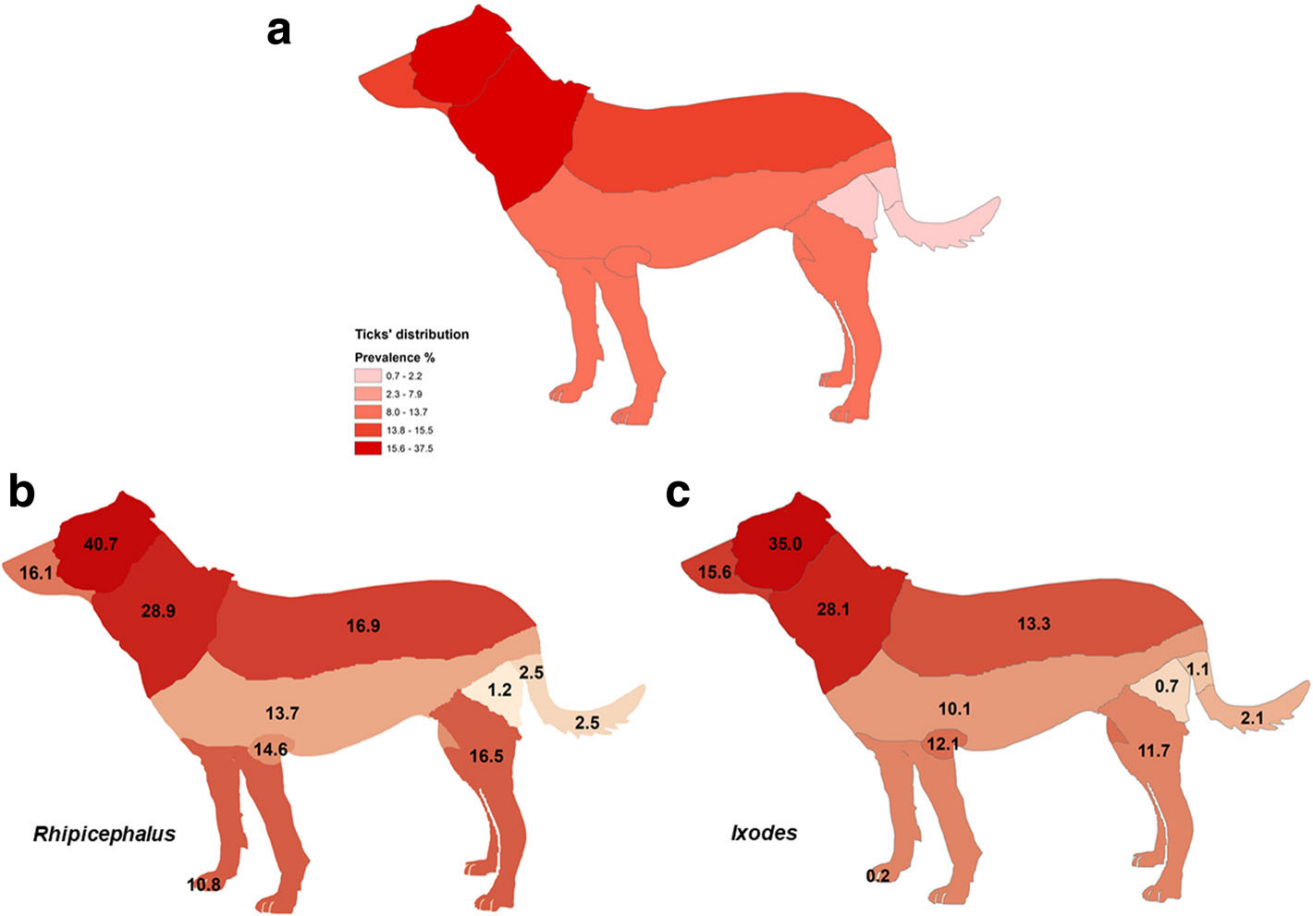
Distribution of ticks on the bodies of dogs enrolled in an Italian survey: **a** total ticks collected; **b** *Rhipicephalus* spp.; and **c** *Ixodes* spp. (data elaborated with ArcGIS 10.3)

Of these, 1383 dogs (45.7%, 95% CI: 43.9–47.5%) were carrying at least one tick; however, ticks were submitted for only 1217 dogs. The body surface was divided in 11 areas and ticks were located on the head (37.4%), the neck (28.8%), the muzzle (15.5%) and the back (15.3%) (Fig. 2a). No significant differences were found either between the overall tick preference for location of attachment on dogs or between the different tick genera regarding their location of preference on the dog’s body. However, a higher prevalence of *Rhipicephalus* spp. was found in interdigital spaces (10.8%) compared to *Ixodes* spp. (0.2%) (Fig. 2b).

**Fig. 3:**
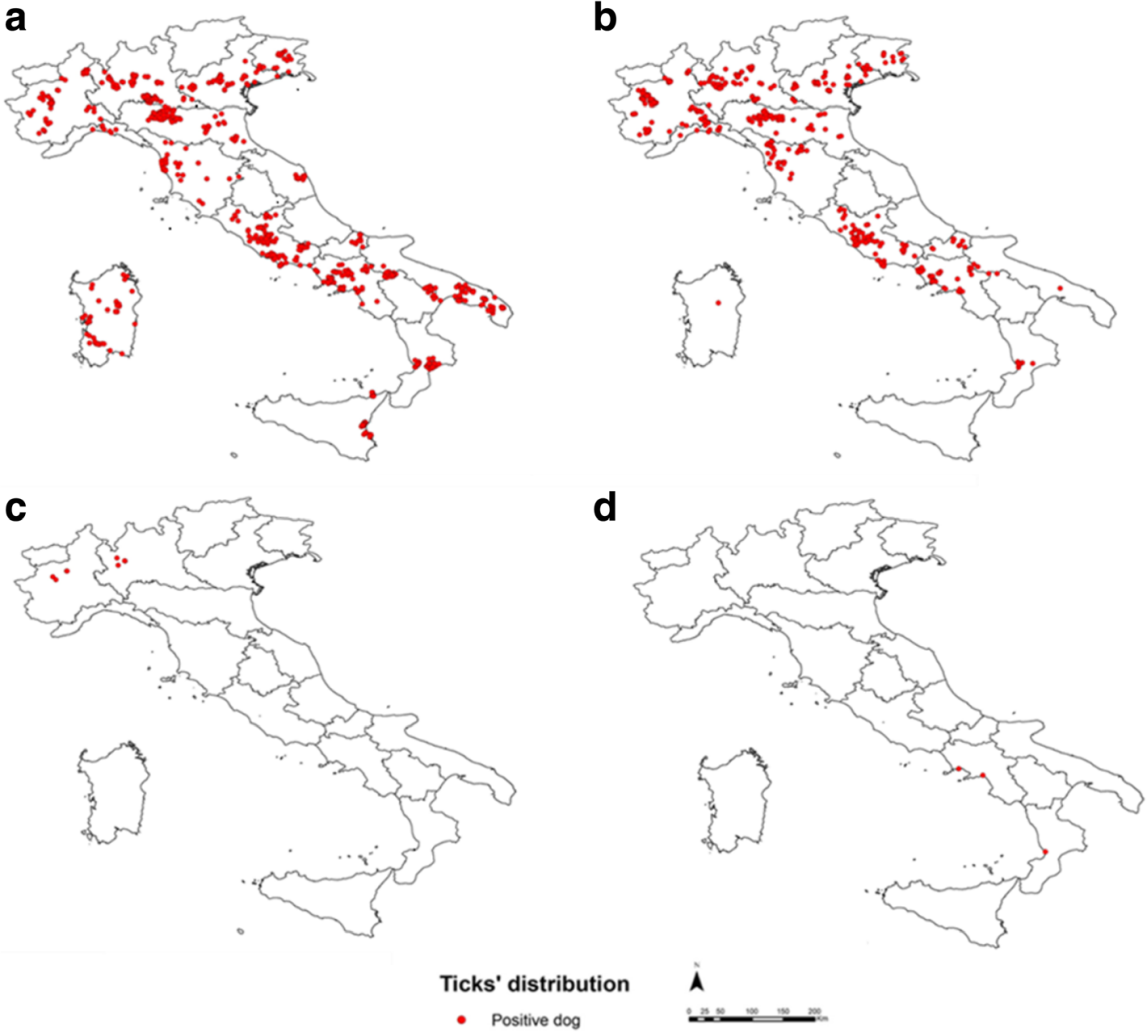
Point distribution maps of four tick genera: **a** *Rhipicephalus*; **b** *Ixodes*; **c** *Dermacentor*; and **d** *Haemaphysalis*, identified in owned dogs surveyed in Italy (2016–2017) (data elaborated with ArcGIS 10.3)

A total of 2439 tick specimens were collected. The median number of ticks per dog was 1, although the number of ticks per animal ranged between 1–44. Specifically, 58 (2.4%) larvae, 325 (13.3%) nymphs and 2056 (84.3%) adults (1373 females and 683 males), belonging to four genera and 14 species (Fig. 3 and Table 1), were found. *Rhipicephalus* and *Ixodes* were the most prevalent genera on dogs in Italy, with *Rhipicephalus* spp. on 27.5% of dogs in the north and 36.1% of dogs in the central-southern regions while *Ixodes* spp. were found on 25.6% of northern dogs and 10.8% of central-southern region dogs. Very few dogs were infested with *Dermacentor* spp. (0.6%) or *Haemaphysalis* spp. (0.2%) ticks. *Rhipicephalus sanguineus* group was most predominant (63.6%), followed by *Ixodes ricinus* (30.6%) and *I. hexagonus* (5.6%) (Table 2). A total of 14 species of ticks were identified showing a different pattern of distribution across Italy (Table 3). Mixed infestations with more than one tick species were recorded on 24 dogs (Table 4).

**Table 1:** Tick species collected from privately owned dogs in Italy classified by life-stage and sex (larvae, nymphs, females and males)

Tick group/species	Larvae	Nymphs	Adults		Total no. of specimens
			Females	Males	
*Rhipicephalus sanguineus* group^a^	21	295	881	625	1822
*Ixodes ricinus*	5	18	399	46	468
*Ixodes hexagonus*	0	7	73	3	83
*Dermacentor marginatus*	0	0	4	1	5
*Rhipicephalus bursa*	0	1	7	3	11
*Dermacentor reticulatus*	0	0	2	5	7
*Haemaphysalis punctata*	0	2	2	0	4
*Ixodes arboricola*	32	0	0	0	32
*Ixodes canisuga*	0	2	2	0	4
*Ixodes gibbosus*	0	0	2	0	2
*Ixodes festai*	0	0	1	0	1
Total	58	325	1373	683	2439

^a^This group includes *Rhipicephalus sanguineus* (*sensu lato*), *R. pusillus*, *R. turanicus* and *Rhipicephalus* sp. I specimens (data confirmed by molecular analysis)

**Table 2:** Prevalence of tick infestation on dogs surveyed in Italy classified by tick species

Tick species	Number of dogs	Prevalence (%)^a^	95% CI
*Rhipicephalus sanguineus* group	769	63.6	60.40–65.89
*Ixodes ricinus*	372	30.6	28.00–33.26
*Ixodes hexagonus*	68	5.6	4.39–7.07
*Rhipicephalus. bursa*	7	0.58	0.25–1.24
*Dermacentor marginatus*	5	0.41	0.15–1.02
*Dermaceontor reticulatus*	3	0.25	0.06–0.78
*Haemaphysalis punctata*	3	0.25	0.06–0.78
*Ixodes arboricola*	2	0.16	0.03–0.66
*Ixodes canisuga*	2	0.16	0.03–0.66
*Ixodes gibbosus*	2	0.16	0.03–0.66
*Ixodes festai*	1	0.08	0.00–0.53

^a^Total number of dogs on which ticks were collected = 1217

**Table 3:** Ixodid species identified in a survey of dogs at veterinary practices in Italy by geographical origin and collection season

Tick species	Geographical origin	Season
*Dermacentor marginatus*	North (Lombardy, Piedmont)	Spring, Summer
*Dermacentor reticulatus*	North (Lombardy, Piedmont)	Spring, Summer
*Haemaphysalis punctata*	South (Calabria, Campania)	Summer, Winter
*Ixodes arboricola*	North (Veneto)	Summer
*Ixodes canisuga*	North (Lombardy)	Summer
*Ixodes festai*	North (Piedmont)	Summer
*Ixodes gibbosus*	South (Calabria, Campania)	Spring
*Ixodes hexagonus*	North (Emilia Romagna, Friuli Venezia Giulia, Lombardy, Piedmont, Veneto), Center (Lazio, Molise, Tuscany), South (Calabria, Campania)	Spring, Summer, Autumn, Winter
*Ixodes ricinus*	North (Emilia Romagna, Friuli Venezia Giulia, Lombardy, Piedmont, Veneto), Center (Lazio, Molise, Tuscany), South (Basilicata, Calabria, Campania, Apulia, Sardinia)	Spring, Summer, Autumn, Winter
*Rhipicephalus* sp. I	South (Basilicata, Apulia)	Spring, Summer
*Rhipicephalus bursa*	Center (Lazio), South (Calabria, Campania, Sicily)	Summer, Winter
*Rhipicephalus pusillus*	Center (Lazio), South (Basilicata, Calabria, Campania, Apulia)	Spring, Summer, Autumn, Winter
*Rhipicephalus sanguineus* (*sensu lato*)	North, Center, South (all the regions investigated)	Spring, Summer, Autumn, Winter
*Rhipicephalus. turanicus*	Center (Lazio), South (Basilicata, Sardinia)	Spring, Summer, Autumn, Winter

**Table 4:** Dogs in a tick survey in Italy infested with a mixed tick infestations

Tick species	Number of dogs
*Rhipicephalus sanguineus* group *+ Ixodes ricinus*	10
*Rhipicephalus sanguineus* group *+ Ixodes hexagonus*	5
*Ixodes ricinus + Ixodes hexagonus*	5
*Rhipicephalus sanguineus* group *+ Rhipicephalus bursa*	2
*Rhipicephalus sanguineus* group *+ Ixodes canisuga*	1
*Rhipicephalus sanguineus* group *+ Dermacentor reticulatus*	1
Total	24

A clear sequence was obtained for only 80 specimens and then compared with GenBank sequences, namely 37 partial *12S* rRNA and 43 partial *16S* rRNA. The length of the *12S* rRNA and *16S* rRNA gene sequences alignments were of 370 and 330 bp, respectively. This sequence analysis showed 99–100% identity to GenBank sequences of *Rhipicephalus sanguineus* (*sensu lato*) (accession numbers: KU255852, KU255849, KU255848, KU556694, KX553960), *R. turanicus* (accession number: KC243822) and *Rhipicephalus* sp. I (accession number: KC243794). Additionally, *D. marginatus* (accession number: JX051098) and *D. reticulatus* (accession number: JF928493) were 99–100% identity to GenBank sequences. Finally, five partial *16S* rRNA gene sequences were identified as *I. canisuga* (accession number: KY962075) and *I. festai* (accession number: KU170522).

### Statistical analysis

The Chi-square test showed that hair length was significantly associated with tick presence (*χ*^2^ = 5.07, *df* = 1, *P* = 0.024). Dog activity and habitat also influenced tick infestation with outdoor (*χ*^2^ = 175.3, *df* = 1, *P* < 0.0001) and rural/sylvatic (*χ*^2^ = 287.1, *df* = 1, *P* < 0.0001) dogs showing higher prevalence.

### Seasonality

Most samples were received between May 2016 and July 2017, during different seasons (Table 3), with fewer submissions at the beginning and end of the survey period, likely associated with reduced practitioner compliance. The sample collection was homogeneous in different regions throughout the study period.

**Fig. 4:**
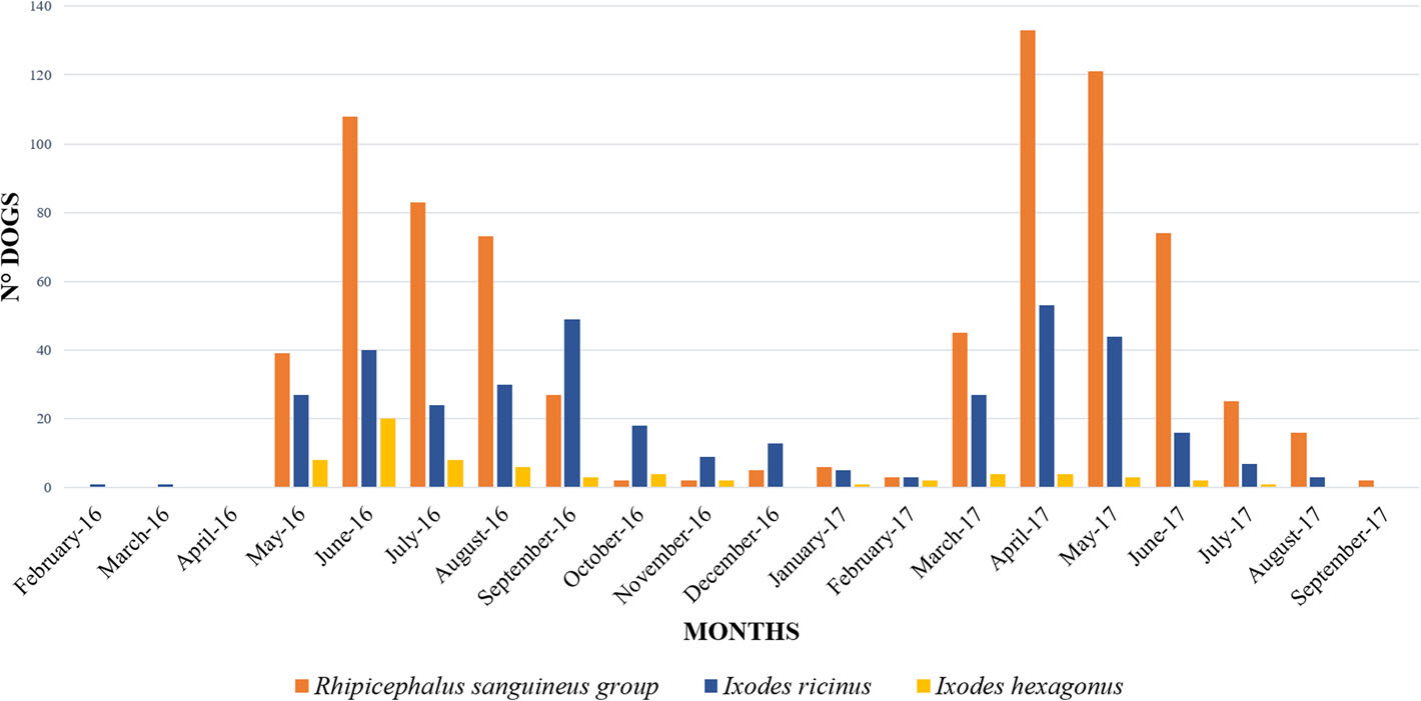
Monthly seasonal dynamics of the three most prevalent ticks (*Rhipicephalus sanguineus* group, *Ixodes ricinus* and *I. hexagonus*) found on owned dogs in a survey in Italy (2016–2017)

More dogs were tick-infested during April to August and the number of tick-infested dogs declined from October to February, although *Rhipicephalus* and *Ixodes* ticks were collected during all months of the year. Ticks of the *Rhipicephalus sanguineus* group showed a peak of infestation during spring and summer while *I. ricinus* and *I. hexagonus* had a lower variation throughout the year (Fig. 4).

### Acaricide treatments

Acaricide treatments were conducted on 2180 dogs (72.0%) in the study, although information on the ectoparasiticide product used for treatment was reported only for 2016 dogs. A total of 687 dogs were not treated with any ectoparasiticide product, while for 159 dogs no information was reported regarding ectoparasiticide use. The majority of treated dogs (1930/2016 dogs; 95.7%) were treated with one product, most frequently a topical spot-on formulation (1278/1930 dogs; 66.2%) followed by oral formulations (348/1930 dogs; 18.0%) and collars (283/1930 dogs; 14.7%). Other formulation types (injectable, spray, shampoo and powder) were each used by < 1% of the screened dogs. Eighty-six dogs were reported to have been treated with a combination of two products. Spot-ons and collars was the most common association, used on 38 dogs.

The ectoparasiticide treatment efficacy was evaluated by comparing tick infestation in untreated dogs to dogs for which information on the product used and the last treatment date was available: (687 untreated dogs and 1320 dogs with complete ectoparasiticide information). Generally, a history of ectoparasiticide treatment was significantly protective against tick infestation (*χ*^2^ = 196.89, *df* = 1, *P* < 0.0001). Differences in the tick control efficacies of oral, spot-on and collar formulations were significant. Considering the recommended retreatment interval for each product and the treatment indications against ticks of the genera *Ixodes*, *Rhipicephalus* and *Dermacentor*, oral formulations provided the highest protection, with 90.1% of dogs tick-free (95% CI: 84.91–93.65%) compared to 69.18% (95% CI: 61.28–76.10%) of dogs tick-free after treatment with antiparasitic collars (oral *vs* collar: *χ*^2^ = 22.87, *df* = 1, *P* < 0.0001) and 53.37% (95% CI: 48.94–57.75%) of dogs were tick-free after treatment with a spot-on acaricide formulation (oral *vs* spot-on *χ*^2^ = 77.08, *df* = 1, *P* < 0.0001). A significant higher efficacy was recorded for spot-on formulations compared to collars (*χ*^2^ = 11.46, *df* = 1, *P* < 0.001). Thirty-two different commercial products were used on dogs enrolled in the study for a total of 13 different active compounds (or active compound combinations). Only 5 compounds were used on more than 50 dogs with details provided of tick infestation prevalence related to compounds most frequently used on enrolled dogs (Table 5). No significant differences were recorded between compounds by generalized linear model (GLM) (*P* > 0.05); however, the greatest proportion of treated dogs that were tick free was with fluralaner (89.5%). Owner respect for recommended retreatment intervals was greater for collars (95.4% active collars), than for oral (70.8% valid treatments) or spot-on formulations (52.6% valid treatments). However, it was also apparent that dog owners and veterinarians lacked knowledge on acaricidal products because 31 dogs were reported to have been treated with products that have no acaricidal activity.

**Table 5:** Ectoparasiticide compounds used on at least 50 dogs in a survey in Italy, showing the proportion of dogs with ticks

Compound	Dogs without ticks	Dogs with ticks	Total no. of dogs	Infestation prevalence (%)
Deltamethrin	39	25	64	39.06
Fipronil	117	133	250	53.20
Flumethrin - Imidacloprid	61	18	79	22.78
Fluralaner	137	16	153	10.46
Permethrin - Imidacloprid	69	55	124	44.35

## Discussion

To our knowledge, this is the first survey on ixodid tick prevalence and distribution on privately owned dogs across an Italian north-south and west-east transect. This study showed that a national tick prevalence survey can be conducted using voluntary practitioner enrolment, as previously carried out in other countries including Belgium [10], Spain [9] and the UK [12].

The geographical distribution of the four genera of ticks found across Italy in this survey is consistent with climatic and environmental features of the Italian peninsula and with maps of tick distribution in Europe (see maps in [26, 27]). *Rhipicephalus* species are widespread in the Mediterranean area while *Ixodes* spp. and *Dermancentor* spp. are better adapted to cold temperate and/or cold continental climates. The presence of *Haemaphysalis* (*H. punctata*) on dogs living outdoor in rural/sylvatic environments in southern Italy is consistent with this tick as the primary species infesting domestic ruminants in this area [28, 29]. However, identification of 14 tick species confirms the high ixodid diversity [30] in Italy. Overall, *R. sanguineus* group (63.6%) was the most prevalent, with high prevalence especially in the central-southern regions (36.1%). This spatial distribution is associated with the suitable microclimate of the south for the development and maintenance of *Rhipicephalus* spp. [31]. *Rhipicephalus sanguineus* (*s.l.*) is the most prevalent species and this is a concern because it is the vector for various TBDs, including ehrlichiosis, anaplasmosis, and babesiosis. In southern Italy, other species belonging to the genus *Rhipicephalus* were found with much lower prevalence (less than 1%), including *R. bursa*, *R. pusillus*, *R. turanicus* and *Rhipicephalus* sp. I. *R. bursa* and *R. turanicus* were reported in dogs from Basilicata, Campania, Latium, Sicily and Sardinia, regions where these species have been reported in livestock [24, 28, 29, 32–34] and where there is small ruminant farming, as recently hypothesized in Greece [35].

*Ixodes ricinus* was the second most common tick species in Italy (29.2%). Higher prevalence values were recorded in the northern regions (20.9%), but this tick was also found in central-southern Italy with a prevalence of 9.5%. *I. ricinus* is a known vector for Lyme borreliosis and so the prevalence of this tick indicates the potential for this disease across Italy.

After *I. ricinus*, the five most prevalent other species of *Ixodes* were found: *I. hexagonus* (4%), *I. arboricola*, *I. canisuga* and *I. festai* (prevalence below 1%) in northern Italy, and *I. hexagonus* (1.2%) and *I. gibbosus* (0.2%) with low prevalence in southern regions.

The general spatial distribution of ticks in Italy observed in the present survey is consistent with findings of large-scale studies conducted in Mediterranean countries including Spain [9], Cyprus [13] and Greece [35]. These studies found *R. sanguineus* group to be the most prevalent tick species, while studies in temperate European countries including the Netherlands [36], Belgium [10] and UK [12] reported a higher prevalence of *I. ricinus* and *I. hexagonus*.

Further studies are needed to clarify ecological preferences for each detected tick species with respect to varying environmental and climatic features of the Italian peninsula and host availability.

The high prevalence of tick-infested dogs (45.7%) in the present study should be carefully interpreted due to a possible over-reporting bias by practitioners as previously hypothesized in a large-scale UK survey [12]. Mixed infestation with two different tick species was found in 24 dogs and is thus quite rare as also reported in Spain [9] and in Greece [35]. In this study, a high number of ticks were adults as observed in other studies [12, 35] which could be a result of difficulties in detecting smaller tick life-stages (larvae and nymphs) during clinical examination [12].

Most ticks were attached to the head, the neck and the thorax/abdomen areas of the dog, and these are more exposed sites for tick attachment as previously reported [10, 37]. The difficulty for dogs to groom ticks from these areas, as well as the skin thickness and local odors could explain this multifocal distribution [31, 37]. Most ticks found in interdigital spaces were *R. sanguineus*, confirming this attachment site as a favorite for this species [38, 39]. This result highlights the importance of systemic acaricidal treatments compared to topical ones because it is unlikely that topically applied and externally acting products will consistently reach acaricidal concentrations on distal locations such as the foot.

Results of our study showed that hair length and lifestyle (indoor *vs* outdoor; urban *vs* rural) were significant tick infestation predictors. A higher number of tick-infested dogs had long hair, possibly because of the greater difficulty for the owner to see and collect the ticks but also because long hair is easier for questing ticks to grab. This is consistent with one previous study [39] but is in contrast with another study [40] that found short-haired dogs significantly more likely to be in the highest infestation category compared with long-haired dogs. Finally, dogs living in rural/sylvatic environments or outdoor were more often tick-infested than pets living in urban areas or indoors as was also recently reported in Greece [35], although *R. sanguineus* (*sensu lato*) was the main group in both countries. No effect of age and sex was found on tick infestation. Previous reports also found that tick presence strongly correlated with tick exposure rather than any specific dog characteristic [12, 40]. Overall, our analysis of the association between tick prevalence and dog characteristics further confirms the inconsistency of this relationship seen among prior tick risk surveys [12].

Seasonal tick species distribution in Italy showed considerable variation between genera. *Rhipicephalus sanguineus* group was most predominant during the spring and summer with an activity peak between April and August. In contrast, *I. ricinus* and *I. hexagonus* were found during all seasons, as observed in other European countries [9, 41–44].

The tick infestation risks for dogs in Italy observed in this study illustrate the importance of years round effective acaricidal treatment. Many owners are already treating their dogs with ectoparasiticidal compounds of a wide variety and types of formulation. Orally administered formulations showed the greatest efficacy; however, no currently available acaricidal treatment can completely prevent tick infestation and tick-borne diseases transmission. In this study, *Rhipicephalus* ticks were found to prefer the interdigital spaces, a difficult location for topical treatments to reach. This is consistent with the suggestion that systemic acaricidal treatment may offer better tick protection than externally distributed treatments. An additional possibility may be to consider combination therapy with both systemic and topically distributed actives [45].

The European Scientific Counsel for Companion Animal Parasites (ESCCAP) in Italy provides quality online information and advice on tick prevalence, tick treatments and tick borne pathogen risk (http://www.esccap.it). Veterinarians and pet owners should make more use of this information, for example to address gaps in their ectoparasiticidal product knowledge (which products are effective against ticks, and which are not). As seen in the results of this survey, several responses indicated that dogs at risk of tick infestation were treated with compounds that did not have an acaricidal effect. This may be due to the fact that most of the ectoparasiticidal products are sold “over the counter” facilitating the availability by owner but at the same time leading to an improper use of the drug.

The importance of using an effective treatment needs to be stressed, and communications from the ESCCAP website, including the parasite control guidelines, provide a reliable tick control information resource.

## Conclusions

This first nationwide survey of ixodid ticks on companion animals in Italy has provided a comprehensive spatial understanding of tick distribution and species abundance, showing that different tick species parasitize dogs in this country. Risk factors vary by dog phenotype and lifestyle. Moreover seasonal species distribution showed considerable variation between tick genera. Further investigations are required to clarify the environmental and host factors that influence tick species infestations on companion animals, in order to develop and plan effective control measures.

## Abbreviations

DF: Degrees of freedom
ESCCAP: European Scientific Counsel for Companion Animal Parasites
EURNEGVEC: European Network for Neglected Vectors and Vector-Borne Infections
GLM: Generalized linear model
TBD: Tick-borne disease
